# Effects of the ridge mulched system on soil water and inorganic nitrogen distribution in the Loess Plateau of China

**DOI:** 10.1016/j.agwat.2018.03.027

**Published:** 2018-04-30

**Authors:** Rui Jiang, Xiao Li, Wei Zhu, Kun Wang, Sheng Guo, Tom Misselbrook, Ryusuke Hatano

**Affiliations:** a Key Laboratory of Plant Nutrition and the Agri-environment in Northwest China, Ministry of Agriculture, College of Resources and Environment, Northwest A&F University, Yangling, 712100, China; b Department of Sustainable Soils and Grassland Systems, Rothamsted Research, North Wyke, Devon, Okehampton, EX20 2SB, UK; c Graduate School of Agriculture, Hokkaido University, Sapporo, 060-8589, Japan; d Zhongshan Torch polytechnic, Zhongshan, Guangdong, 528436, China

**Keywords:** Soil water, Inorganic N, Plastic film mulch, Ridge mulched system, Maize, Loess Plateau

## Abstract

The semi-arid region of the Loess Plateau is typical of rain-fed agricultural production in Northwestern China. In this area, the ridge mulched system (RM) is a widely-used measure to increase crop yield. The purpose of this study was to investigate the effect of RM on soil water and inorganic nitrogen (N) distribution, and grain yield of maize (*Zea mays* L.). The study was conducted over three consecutive years and consisted of four treatments (each replicated three times): i) RM with N application rate of 260 kg N ha^−1^ (RM-N260); ii) RM with 180 kg N ha^−1^ (RM-N180); iii) a traditional flat cultivation system without mulching (F) with 260 kg N ha^−1^ (F-N260); iv) F with 180 kg N ha^−1^ (F-N180). Mean soil water content during the maize growing season was increased by RM in 2013 only. However, RM increased the soil water storage significantly at the 3-leaf (V3) and 6-leaf stage (V6), and decreased evapotranspiration (ET) during pre-silking stage in all years. Compared to F, RM significantly improved maize grain yield by 79–123% in 2013, 23–25% in 2014, and 11–12% in 2015. Following three years of maize cultivation, soil inorganic N content increased substantially (two- to three-fold) in the RM system and 60% of the total inorganic N was accumulated in the top soil layers (0–60 cm) under the mulched ridge. Relative changes were much smaller in F, and most of inorganic N was stored in 0–20 cm and 100–160 cm soil layers. Generally, RM resulted in higher soil water storage during the pre-silking stage, which was the main reason for the improved maize grain yield. The nitrate leaching risk was reduced in RM-N180 compared with F, but nitrate leaching from the furrows between ridges was observed in RM-N260. However, the large increase in soil inorganic N content in RM-N180 after three years’ cultivation indicates an oversupply of N and a potential risk of N losses to the environment over the longer term. Our study indicates, therefore, that RM is a suitable system for maize cropping in the semi-arid region of the Loess Plateau, with benefits in water and N use efficiency, but recommendations for appropriate N application rates are required to ensure long term agricultural sustainability, accounting for grain yields and environmental impacts. The mechanisms for inorganic N accumulation under the RM system are not fully understood and warrant further investigation.

## Highlights

• We evaluated the effect of a ridge mulched system (RM) on soil water and inorganic N.
• RM increased soil water storage and decreased evapotranspiration during the pre-silking stage.
• RM increased soil inorganic N in the topsoil under the ridge and reduced N leaching.
• RM led to high inorganic N accumulation in soil after three years maize cultivation.

## 1 Introduction

Plastic film mulching has developed rapidly following it’s introduction to China in 1978 and is now widely applied in crop production in arid and semiarid regions (Dong et al., 2009; Li et al., 2004). Several mulching systems have been used in recent years, including 1) fully mulched ridge and furrow system: two ridges and furrow fully mulched with plastic film (Zhou et al., 2009); 2) ridge mulched system: alternating ridge and furrow with plastic film mulched-ridge (Liu et al., 1989); 3) flat half mulched system: alternating mulched row and bare row in flat cultivation (Liu et al., 1989); and 4) flat fully mulched system: flat plot all mulched with plastic film (Liu et al., 1989). Of these, the ridge mulched system has been most widely adopted, leading significant increases in crop yield rain-fed agricultural areas of Chinese Loess Plateau, especially in areas with 400–600 mm annual precipitation (Jiang et al., 2016; Wang et al., 2015; Wang et al., 2016). The yield increases under plastic film mulching have been attributed to factors including: 1) reduction in soil evaporation and increase in crop transpiration; 2) increase in water harvesting; 3) increase in soil temperature; and 4) increase in activation of soil nutrients (Zhou et al., 2009; Zhao et al., 2002; Zhou, 1996). These factors change the soil environment, improving conditions for crop growth, resulting in higher water use efficiency and nutrient availability.

Water is the most limiting factor for crop production under rain-fed agriculture in arid and semiarid areas. However, studies have shown that the temporally irregular rainfall distribution and inefficient management of rainwater, rather than total rainfall amount, are the primary limits for crop production (Barron et al., 2003; Zhu et al., 2015). Zhu et al. (2015) indicated that plastic film mulching improved rainwater management to overcome water limitations during dry spells, which promoted crop growth and increased yields significantly. Drought and mild chilling stress often occur in the Loess Plateau of China, especially during the early stage of the maize growing season; plastic film mulching can improve the soil water content and temperature during this stage resulting in increased yield (Jiang et al., 2016; Zhang et al., 2014). The main crops (i.e. spring maize and winter wheat) on the Loess Plateau are conventionally cultivated as a single crop per year followed by several months of bare fallow. It is generally assumed that water stored in the soil during fallow period is utilized by the subsequent crop. The antecedent soil moisture content (prior to sowing) may also cause or mitigate water stress during the early stages of crop growth. However, few studies have considered the interactions between plastic film mulching and antecedent soil moisture content on crop yield.

Fertilization can increase crop yield and enhance drought resistance of crops in arid and semiarid regions (Liu et al., 2013). However, excessive application of N fertilizer results in low N use efficiency and high N losses with environmental impacts including greenhouse gas emission, water contamination, soil quality degradation, and soil nitrate accumulation in deep soil layers (Davidson 2009; Morell et al., 2011; Reay et al., 2012; Zhou et al., 2016). Plastic film mulching not only results in improved soil physical properties (soil water and temperature) but also directly changes soil biological characteristics and soil fertility (Liu et al., 2013). Soil inorganic nitrogen (N) is an important indicator for soil fertility and productivity. Since the plastic film mulching changes the soil microenvironment with associated changes to the N cycle processes, the soil inorganic N distribution under plastic film mulching systems may differ to that under traditional cultivation. Plastic film mulching may increase soil microbial activity due to the improved soil water and temperature conditions and thereby enhance mineralization (Wang et al., 2006). High inorganic N contents have been observed in soil profiles under plastic film mulching, especially in topsoils, and enhanced soil mineralization could be one reason (Wang et al., 2006). Another reason may be related to reduced N leaching under plastic film mulching (Ruidisch et al., 2013; Liu et al., 2017a, Liu et al., 2017b). However, Liu et al., 2014a, Liu et al., 2014b found that the nitrate accumulation was more related to N fertilizer application rates. High N input resulted in higher nitrate accumulation in the soil profile, which may also increase N leaching and greenhouse gas emissions (Kettering et al., 2013; He et al., 2018; Anikwe et al., 2007). Several studies have investigated the fate of N under plastic film mulching, however, few have considered the potential differences that may occur between the ridge and furrow under a ridge mulched system in arid and semiarid areas (Liu et al., 2015; Wang et al., 2016; Kettering et al., 2013). Ridge mulched systems have a mulched ridge and furrows without mulch, thus the N transformations and transport processes could be different. A study conducted under a monsoon climate showed that N leaching mainly occurred from the furrow in a ridge mulched system (Kettering et al., 2013). However, the vertical distribution and temporal patterns of soil inorganic N under ridge mulched systems are not well understood in rain-fed drylands.

Many studies have investigated the relationship between soil water content and crop yield to clarify the yield increase mechanism under plastic film mulching, however, very few have considered the impacts of the effects on water content and soil inorganic N distribution on both food security and sustainable agriculture. This study focused on a ridge mulched system in a maize crop on the Loess Plateau of China, taking crop production and environmental sustainability into consideration. The objectives were to 1) evaluate the effect of the ridge mulched system on soil water content, soil water storage, and water use efficiency; and 2) investigate soil inorganic N pools and distribution, considering the ridge and furrow as different units.

## 2 Material and methods

### 2.1 Site description

The field experiments were conducted from 2013 to 2015 at the Changwu Agricultural and Ecological Experimental Station, located on the Loess Plateau of China (35.28°N, 107.88°E, ca. 1200 m above sea level). The annual mean air temperature is 9.2 °C and average annual rainfall is 582 mm, 73% of which occurs during the maize-growing season. The groundwater table is approximately 80 m below the surface. The cropping system in this area is one crop of maize or wheat per year. According to Chinese Soil Taxonomy, the soils are Heilutu, belonging to Cumuli-Ustic Isohumosols (light silt loam) (Gong et al., 2007). The soil properties in the top 20 cm are as follows: bulk density, 1.3 g cm^−3^; pH, 8.4; soil organic carbon content, 9.5 g kg^−1^; total N content, 1.05 g kg^−1^; available phosphorus (Olsen-P) content, 20.7 mg kg^−1^; available potassium (NH_4_OAc-K) content, 133.1 mg kg^−1^; and mineral N content, 28.8 mg kg^−1^.

### 2.2 Field experiments

**Fig. 1:**
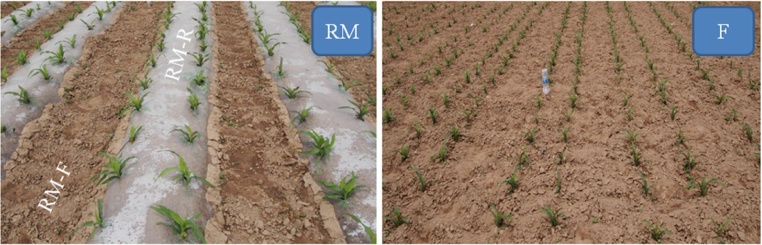
Maize cultivation systems used in the study. Left picture shows the ridge mulched system (RM) (RM-R: ridge in ridge mulched system; RM-F: furrow in ridge mulched system); right picture shows the flat cultivation system (F).

This study included a ridge mulched system (RM) and a traditional cultivation system (i.e. flat cultivation without mulching) (F) over three maize-growing seasons (Fig. 1). There were two fertilizer application treatments: 180 kg N ha^−1^, as recommended for traditional cultivation; 260 kg N ha^−1^ as per local farmer practice, giving a total of four treatments (RM-N180, F-N180, RM-N260, and F-N260). Plots were established a 5 × 10 m in a randomized block design with three replicate plots per treatment. Urea (N 46%) was used as N fertilizer in all fertilized treatments. In 2014 and 2015, all N fertilizer was applied manually to the soil surface prior to sowing and plowed in as a basal dressing. In 2013, 70% of the N fertilizer was applied as basal dressing and the remaining 30% was applied during the silking stage using a hole-sowing machine following precipitation. A total of 60 kg P ha^−1^ as calcium superphosphate (P_2_O_5_ 12%) and 75 kg K ha^−1^ as potassium sulfate (K_2_O 45%) were applied simultaneously with the basal N fertilizer for each plot. Ridges and furrows were made following fertilizer application.

**Fig. 2:**
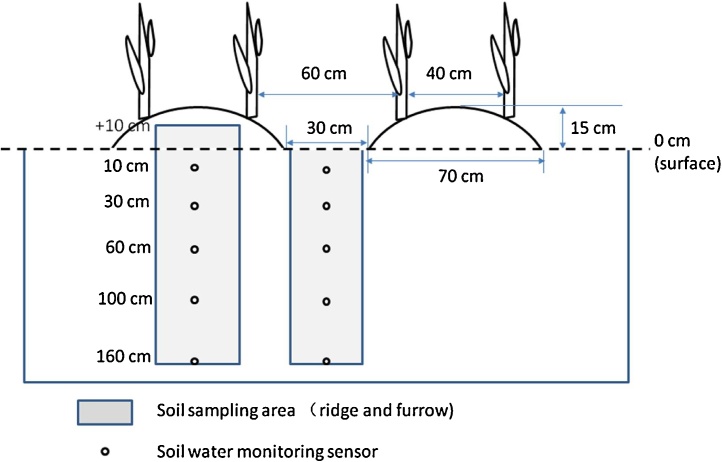
Schematic of the RM system and the locations for soil sampling and soil water monitoring.

The RM system consisted of alternating ridges (70 cm) and furrows (30 cm), with only the ridges being mulched with plastic film (Fig. 2). Two rows of maize were planted with a 40 cm spacing centered on the 70 cm wide ridges. Thus the RM system contained alternating wide (60 cm) and narrow (40 cm) plant row spacings. The F system also had the identical wide and narrow row spacings. Within-row plant spacing was 30 cm, and the plant density was approximately 67,000 plants ha^−1^, typical of local practice. The plastic film in all treatments was 0.008-mm-thick transparent polyethylene. A high-yielding maize hybrid (Pioneer 335) was selected for this study. The maize was planted at the end of April and harvested at the end of September. There was no irrigation during the maize-growing season.

### 2.3 Monitoring and sampling

The monitoring and sampling was conducted during the maize growing seasons from April 2013 to September 2015. The soil water monitoring was conducted separately for ridges and furrows in the RM (RM-R and RM-F) and F treatments (Fig. 2). The soil water content (volumetric water content) was measured using soil moisture monitoring instruments (ECH_2_O System, Decagon, USA) at 1 h intervals during 2013 with 1 h interval. Six ECH_2_O systems were installed and each consisted of five ECH_2_O-TE sensors, which were installed at soil depths of 10, 30, 60, 100, and 160 cm, to derive soil water contents at the 0–20, 20–40, 40–80, 80–120, and 120–200 cm layers. Sensors were calibrated before installation and the soil moisture at each depth in the four soil profiles was assumed as homogenous before the experiment (details given in Jiang et al., 2016). Soil moisture content was determined gravimetrically at the same measured depths for another two replicates for each treatment at 15 day intervals using a soil auger. In 2014 and 2015, soil water content (N = 3) was measured using a Neutron Moisture Meter (CNC503DR) every 15 days at the same measured depths as in 2013. At the same time, soil water content at depths of 10 and 30 cm was determined gravimetrically using a soil auger. The ECH_2_O-TE sensor and neutron probe were thus calibrated against gravimetrically measured soil water contents.

Soil samples (RM-R: +10–0, 0–10, 10–30, 30–60, 60–100, 100–160 cm, RM-F and F: 0–10, 10–30, 30–60, 60–100, 100–160 cm; 3 replicates per plot) were collected at 15 day interval during maize growing season with a soil corer (diameter: 5 cm). Soil samples were refrigerated at 4 °C prior to subsequent analysis. The soil nitrate-N (NO_3_^−^-N) and ammonium-N (NH_4_^+^-N) concentrations were measured using a continuous flow analyzer (AA3, Seal Analytical, Germany) after KCl extraction. The nitrite-N (NO_2_^−^-N) was ignored in this study, because most samples were lower than detection limits. We used the NO_3_^−^-N and NH_4_^+^-N concentrations in dry weight equivalent soil and bulk density to calculate the inorganic N pools in kg ha^−1^ for the different depths. For RM, the soil water storage (SWS) and inorganic N pools were calculated by using the respective data for RM-R and RM-F and multiplying by the ridge to furrow ratios (ridge ratio: 7/10; furrow ratio: 3/10).

### 2.4 Statistical analysis

The mean and standard deviation were calculated for the data collected for each treatment and depth. The differences between treatments and depths were analyzed using one-way analysis of variance (ANOVA) and the least significant difference (LSD) was used for multiple comparisons. Differences were considered statistically significant at P < 0.05.

## 3 Results and discussion

### 3.1 Soil water content, soil water storage, and water use efficiency during maize growing season

Compared with the long-term average (1957–2009), the annual precipitation was 68.6, 170.0, and 63.8 mm lower in 2013, 2014, and 2015, respectively (Table 1). The precipitation during the maize growing season for the three years accounted for 85, 67 and 67% of the annual precipitation, which was 17 mm higher, 142.4 mm and 73.6 mm lower the than long-term average for 2013, 2014 and 2015, respectively. The E601 pan evaporation was lower in all years than the long-term average for both annual and during the maize growing season. The precipitation during one month and three months before sowing were lowest in 2013, approximately 20 and 43 mm lower than long-term average; however, the evaporation for those periods was higher than usual. The drought situation started before maize sowing and lasted to the end of June (pre-silking stage) in 2013 (Table 1). In 2014, there was higher precipitation and lower evaporation during one month and three months before sowing.

**Table 1:** The climate characteristics (precipitation: P and E601 pan evaporation: Evaporation) during 2013–2015.

	2013		2014		2015		Long-term average (1957–2009)	
	P (mm)	Evaporation (mm)	P (mm)	Evaporation (mm)	P (mm)	Evaporation (mm)	P (mm)	Evaporation (mm)
Annual	515.4	832.0	414.0	748.0	520.2	855.2	584.0	892.0
Growing season	437.0	461.6	277.6	477.9	346.4	488.8	420.0	557.4
Pre-silking	44.8	218.6	85.2	195	149	176.4	–	–
One month before sowing	21.8	111.8	83.4	44.0	57.4	69.5	41.2	101.3
Three months before sowing	29.6	261.1	118.0	123.8	89.8	213.1	72.5	203.4

Significant differences between treatments in mean soil water content across all soil layers were only observed in 2013, although some soil layers were also shown the differences in 2015 (Table 2). In 2013, the soil water content was 10% lower in RM-R than that in F at 10 cm depth, while 20% higher in RM-F. The RM significantly increased the soil water content at depths of 30 and 60 cm, by 40 and 22% at 30 cm and 43 and 47% at 60 cm for RM-R and RM-F, respectively. In addition, the mean soil water contents were the lowest in 2013 across the three years for each soil layer and each treatment. The extreme drought situation before sowing lasted to end of June and is the most likely reason for the observed lower soil water content in 2013 (Table 1). This was supported by the significantly lower maize yields in 2013 for F treatments (Table 3), which showed that the drought limited maize growth in 2013 but the soil water content was sufficient for maize growth in 2014 and 2015. However, the use of different measurement methods for monitoring soil water contents between 2013 and 2014/2015 may also have contributed to these observed differences, although both methods were calibrated against gravimetrically measured soil water contents.

**Table 2:** Soil water content in different soil layers in different treatments (RM-R-N260 and RM-R-N180: ridge in ridge mulched system at N application rate of 260 and 180 kg N ha^−1^; RM-F-N260 and RM-F-N180: furrow in ridge mulched system at N application rate of 260 and 180 kg N ha^−1^; F-N260 and F-N180: flat cultivation system at N application rate of 260 and 180 kg N ha^−1^).

	+10 cm		0–10 cm		10–30 cm		30–60 cm		60–100 cm		100–160 cm	
	Average	Std	Average	Std	Average	Std	Average	Std	Average	Std	Average	Std
2013												
RM-R-N260	0.189	0.051	0.138 d	0.035	0.219 a	0.058	0.190 c	0.024	0.193 ab	0.039	0.178 b	0.005
RM-F-N260			0.188 a	0.051	0.190 b	0.111	0.195 bc	0.040	0.198 a	0.050	0.180 b	0.028
F-N260			0.151 bc	0.077	0.156 c	0.046	0.144 d	0.031	0.197 a	0.047	0.204 a	0.034
RM-R-N180	0.167	0.056	0.144 dc	0.041	0.214 a	0.069	0.201 ab	0.030	0.185 b	0.028	0.165 c	0.023
RM-F-N180			0.186 a	0.059	0.188 b	0.059	0.208 a	0.033	0.201a	0.029	0.157 d	0.019
F-N180			0.162 bc	0.059	0.153 c	0.049	0.131 e	0.037	0.185 b	0.071	0.197 a	0.024

2014												
RM-R-N260	0.187a	0.057	0.198a	0.058	0.259a	0.089	0.244a	0.072	0.219a	0.059	0.263a	0.067
RM-F-N260			0.211a	0.068	0.260a	0.097	0.243a	0.065	0.217a	0.056	0.261a	0.050
F-N260			0.183a	0.075	0.243a	0.088	0.225a	0.074	0.227a	0.063	0.238a	0.042
RM-R-N180	0.179a	0.050	0.202a	0.060	0.270a	0.108	0.265a	0.153	0.224a	0.062	0.251a	0.055
RM-F-N180			0.200a	0.065	0.263a	0.106	0.226a	0.087	0.224a	0.076	0.251a	0.056
F-N180			0.191a	0.070	0.246a	0.091	0.222a	0.075	0.226a	0.068	0.240a	0.047

2015												
RM-R-N260	0.207	0.048	0.229 ac	0.047	0.252	0.044	0.271a	0.118	0.232a	0.038	0.239 a	0.026
RM-F-N260			0.238 a	0.041	0.243	0.049	0.220 b	0.036	0.235a	0.041	0.239 a	0.028
F-N260			0.208 ac	0.045	0.236	0.047	0.236 ab	0.036	0.227a	0.030	0.227 ac	0.017
RM-R-N180	0.213	0.034	0.226 ac	0.035	0.270	0.055	0.204 b	0.047	0.214a	0.048	0.214 bc	0.028
RM-F-N180			0.201 c	0.040	0.251	0.047	0.214 b	0.051	0.219a	0.047	0.222 ac	0.027
F-N180			0.202 bc	0.039	0.242	0.038	0.194 b	0.047	0.231a	0.033	0.226 ac	0.020

**Table 3:** The △SWS (changes in soil water storage in the 0–200 cm profile between harvest and sowing), ET (evapotranspiration, ET = Precipitation-△SWS^1^), crop yield and WUE (water use efficiency) of different treatments during maize growing season.

	△SWS (mm)	ET (mm)			Yield (kg ha^−1^)	WUE (kg ha^−1^ mm^−1^)
		Growing season	Pre-silking	Post-silking		
2013						
RM-N260	139.2 a	356.2 a	13.3a	342.9a	13800 a	38.7 a
F-N260	120.7 b	374.7 b	27.4b	347.3b	7700 b	20.5 b
RM-N180	141.9 c	353.5 a	12.2a	341.3a	14700 c	41.6 c
F-N180	123.7 d	371.7 b	44.1c	327.6c	6600 d	17.8 d

2014						
RM-N260	-33.3 a	438.1 a	137.5a	300.6a	16377 a	37.4 a
F-N260	5.1 b	399.7 b	154.6b	245.1b	13366 b	33.4 b
RM-N180	-20.6 c	425.4 a	138.8a	286.6c	16380 a	38.5 a
F-N180	-58.2 d	463.0 c	179.1c	283.9c	13088 b	28.3 c

2015						
RM-N260	-39.8 a	386.2 a	99.4a	286.8a	14270 a	36.9 a
F-N260	-53.4 b	399.8 b	117.5b	282.3a	12702 b	31.8 b
RM-N180	-52.9 b	399.3 b	109.5c	289.8a	14095 a	35.3 a
F-N180	-34.5 a	380.9 a	115.6b	265.3b	12720 b	33.4 b

1 A soil depth of 2–3 m was recommended for ET estimation of annual field maize crop in the Loess Plateau area, because more than 90% of ET normally comes from 0 to 2 m soil layers (Li et al., 1985). A soil thickness between 2 and 3 m should be sufficient for estimating field water balance in the region in years having average or below-average precipitation (Liu et al., 2010), therefore the deep drainage below 2 m was neglected in this study.

**Fig. 3:**
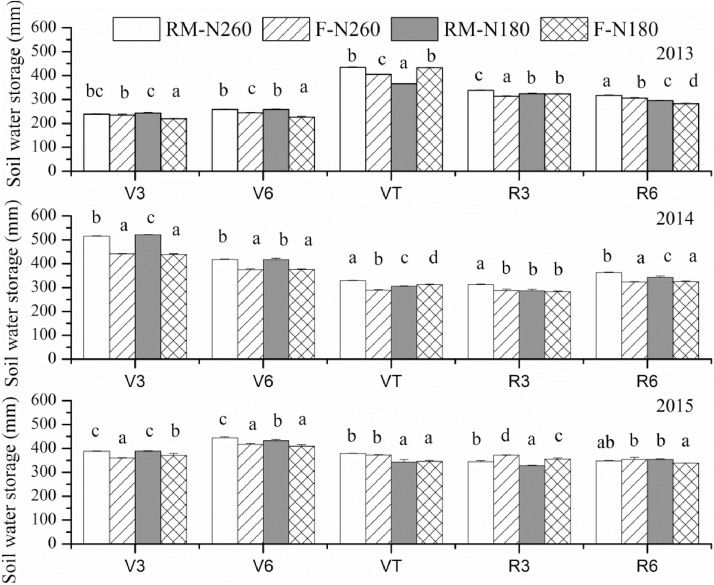
Soil water storage in the 0–200 cm soil profile for different growing stages in RM-N260, F-N260, RM-N180, and F-N180 during the maize growing seasons in 2013, 2014, and 2015. Error bars are the standard deviation of the mean (n = 3). Different lowercase letters indicate significant difference at P < 0.05. V3: 3-leaf stage; V6: 6-leaf stage; VT: silking stage; R3: milk stage; R6: maturity stage. RM-N260 and RM-N180: ridge mulched system at N application rate of 260 and 180 kg N ha^−1^; F-N260 and F-N180: flat cultivation system at N application rate of 260 and 180 kg N ha^−1^.

**Fig. 4:**
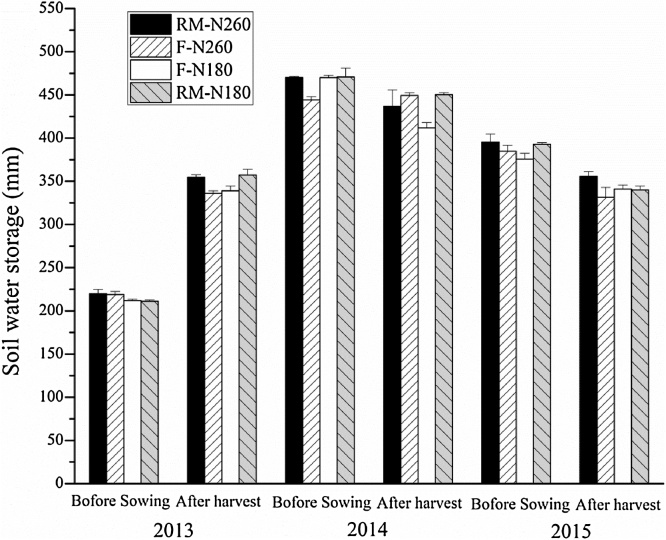
The soil water storage for different treatments before sowing and after harvest.

The RM treatments increased SWS during the early stage of the maize growing season. Compared with the F treatments, the average SWS in the RM treatments was 13, 78, and 23 mm higher at the 3-leaf stage (V3) and 23, 42, and 26 mm higher at the 6-leaf stage (V6) in 2013, 2014, and 2015, respectively (Fig. 3). From the silking stage (VT), the difference between RM and F decreased, and SWS was higher in F following large rainfall events (i.e. VT stage in 2013 and R3 stage in 2015). The SWS at V3 and V6 stage were 277 and 160 mm lower in 2013 than that in 2014, and 150 and 187 mm lower than that in 2015. Allowing for the lower precipitation during the early stage of the maize growing season in 2013, this might also be a result of the much lower SWS before sowing (only 215 mm on average across all treatments), compared with the 464 and 387 mm of SWS before sowing in 2014 and 2015. The SWS in 2013 increased 117–146 mm at harvest for all treatments; however, in 2014 and 2015, with the higher values before sowing, SWS decreased by 27 and 45 mm, respectively, on average across all treatments (Fig. 4, Table 3).

The changes in SWS across the 0–200 cm soil depth between harvest and sowing (△SWS) was significantly higher in RM than that in F in 2013; while the evapotranspiration (ET) was lower in RM (Table 3). No significant difference in ET across the entire growing season was observed between RM and F in 2014 and 2015. However, during the pre-silking stage, ET was significantly lower in RM than in F for all three years and higher in RM during the post-silking stage (Table 3). The effect of plastic film mulching on ET was mainly to change the ratio between soil evaporation and maize transpiration (Li et al., 2013). A higher aboveground biomass after the 6-leaf stage has been observed under plastic film mulching (Jiang et al., 2016), and we observed higher soil water contents in RM before the silking stage (Fig. 3). This suggests that the lower ET during the pre-silking stage was due to lower soil evaporation and the higher ET during the post-silking stage was due to higher plant transpiration in RM. Compared with the F treatments, RM significantly increased maize grain yield (Table 3), with a much greater effect observed in 2013. The yield increases for RM-N260 and RM-N180, compared with F-N260 and F-N180, were 79 and 123% in 2013, 23 and 25% in 2014, and 12 and 11% in 2015, respectively. Several studies have shown that ET (or the contribution of transpiration to ET) and water use during pre-silking stage has a positive relationship with grain yield (Zhang et al., 2014; Zhu et al., 2015), as observed in our study. The highest yields occurred in 2014 both in RM and F, associated with much higher precipitation and lower evaporation before sowing (and a much higher SWS before sowing, Fig. 4) even though precipitation during growing stage was lower (Table 1), implying that the soil water content before sowing plays an important role in maize grain yields. Zhang et al. (2014) also found a significant correlation between crop yield and soil water content at sowing on the Loess Plateau. Zhu et al. (2015) found SWS at sowing had a significant positive relationship with ET during the pre-silking stage. We also observed higher ET during the pre-silking stage associated with higher SWS at sowing (Table 3). Thus, the SWS at sowing might increase plant transpiration during the pre-silking stage and affect grain yield. RM significantly increased WUE, which increased by 89 and 134% for RM-N260 and RM-N180 in 2013, 12 and 36% in 2014, and 16 and 6% in 2015, respectively (Table 3).

These finding suggest that maize grain yield was not limited by the amount of rainfall during the maize growing season or the cumulative annual rainfall but by the irregular distribution of rainfall over time and the inefficient management of rainwater in semiarid areas (Zhu et al., 2015). Many studies have indicated that increasing rainwater harvest, improving soil water storage, and increasing plant water uptake capacity by using different cultivation systems could optimize crop productivity in rain-fed areas (Kahinda et al., 2007; Zhu et al., 2015). In our study, the maize grain yields were significantly higher in RM than that in F treatments (Table 3). Previous studies have found that maize production on the Loess Plateau was constrained by water stress at the early stage of the maize growing season (Liu et al., 2009; Song et al., 2013; Zhang et al., 2013; Jiang et al., 2016), as seen in our study (i.e. low yields in F in 2013). The drought during pre-silking (particularly the jointing stage) postponed silking and shortened the grain filling period, and thus affected final grain yield (Moser et al., 2006). Our results suggested that increases in maize yields with RM were primarily through the improved soil moisture especially during drought periods. Significantly higher soil water storage was observed during the V3 and V6 stages across all three years, which could be explained a reduction in soil evaporation with mulching, enhancing the harvesting of the small rainfall amount (Li et al., 2001), and improving the soil water use efficiency (Table 3). Zhu et al. (2015) found that the higher soil water storage in the early growing season in mulching systems clearly promoted maize growth and development, because the higher soil water storage was sufficient to provide a buffer during drought periods which usually occurred in the early growing season. This could also explain the large increase in maize yield for RM in 2013 in our study. Although the difference in ET for the entire growing season between RM and F was not consistent across all three years, RM actually reduced nonproductive soil evaporation but increased productive plant transpiration during the pre-silking stage as discussed above, thereby improving the water use efficiency and yield (Table 3). In addition, we found that the SWS before sowing could be a key factor for maize production, which could mitigate the water stress during the maize growing season (i.e. high yields in F in 2014 with low annual rainfall). This result indicated that rainfall in the fallow season may play an important role in water use in the next year. Thus, if precipitation during fallow seasons could be effectively harvested and stored in the soil through management intervention (such as using plastic film or leaving straw residue after harvest), the water requirements for maize would be satisfied, which is particularly important for dry years.

### 3.2 Dynamics of inorganic N content in the soil profile during the maize growing season

**Fig. 5:**
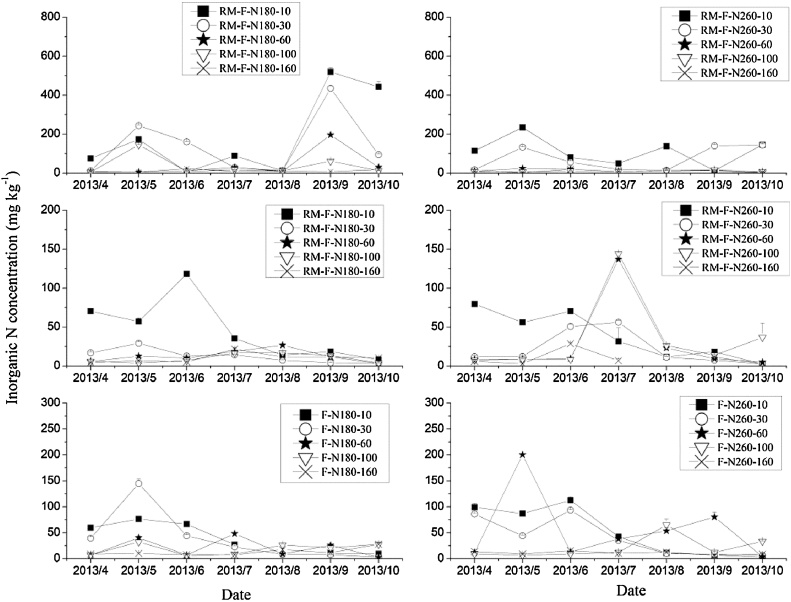
Inorganic N concentrations in different soil layers during the 2013 maize growing season. (RM-R-N260 and RM-R-N180: ridge in ridge mulched system at N application rates of 260 and 180 kg N ha^−1^; RM-F-N260 and RM-F-N180: furrow in ridge mulched system at N application rates of 260 and 180 kg N ha^−1^; F-N260 and F-N180: flat cultivation system at N application rates of 260 and 180 kg N ha^−1^).

**Fig. 6:**
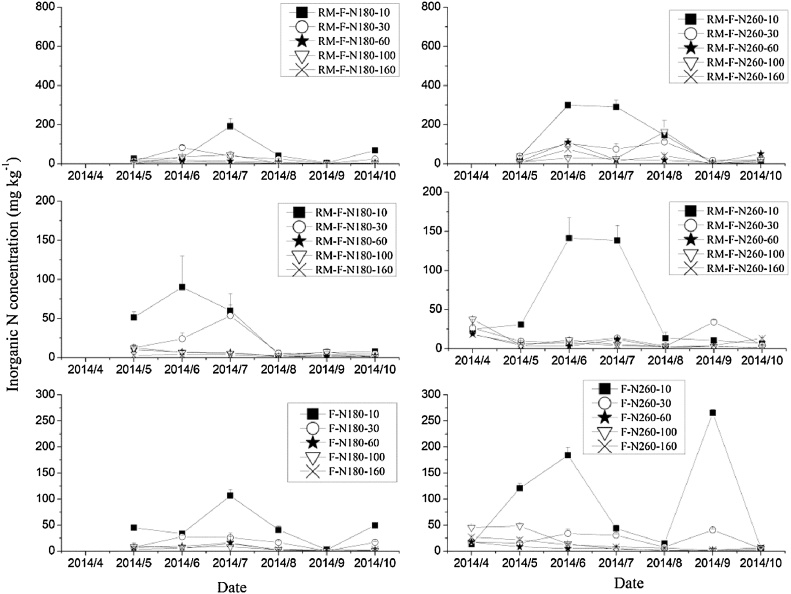
Inorganic N concentrations in different soil layers during the 2014 maize growing season.

**Fig. 7:**
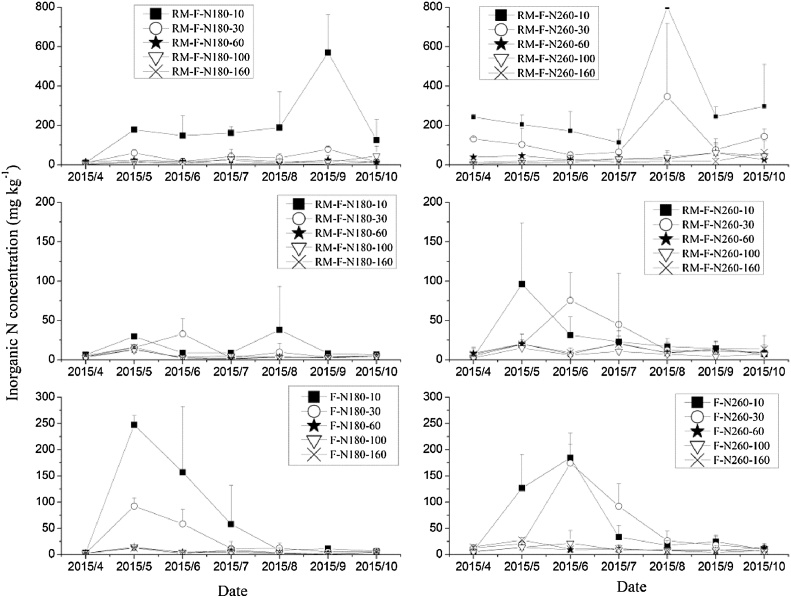
Inorganic N concentrations in different soil layer during the 2015 maize growing season.

The dynamics of soil inorganic N content in the 0–160 cm soil layer during the maize growing season for 2013–2015 in RM and F treatments are shown in Fig. 5, Fig. 6, Fig. 7. There were large fluctuations in soil inorganic N in the top layers (0–30 cm) and the mean contents in these layers were much larger than that in deeper layers. Nitrate-N was the main form of inorganic N, accounting for 63–98% in this study (Table 4). The observed higher nitrate-N contents in the top soil layers is consistent with other studies (Wang et al., 2015; Thorup-Kristensen, 2001).

**Table 4:** Fertilizer application rates, ratio of inorganic N to total N, ratio of nitrate-N to inorganic N, harvest N, and partial factor productivity (PFP) in different systems before and after maize cultivation.

	Fertilizer application rate (kg N ha^−1^)	Inorganic N/TN	NO_3_^−^-N/Inorganic N	PFP
before sowing in 2013		1.7	91.5	
after harvest in 2013				
RM-N260	260	2.8	98.0	53.1
F-N260	260	1.2	94.5	31.7
RM-N180	180	2.1	92.6	75.0
F-N180	180	1.4	91.7	45.6

after harvest in 2014				
RM-N260	260	–	97.9	63.0
F-N260	260	–	96.7	51.4
RM-N180	180	–	80.1	91.0
F-N180	180	–	84.6	72.7

after harvest in 2015				
RM-N260	260	7.8	96.0	54.9
F-N260	260	2.5	95.2	48.9
RM-N180	180	3.5	86.4	78.3
F-N180	180	1.1	62.8	70.7

On average, the inorganic N contents in the 0–10 cm soil layer for RM-R were 1.3–23.4 times and 1.5–13.1 times higher than that in other soil layers for RM-N180 and RM-N260, respectively, across the three years. High levels of inorganic N were mainly distributed in the 0–10 cm layer in the ridge with mulching, as observed in other studies (Wang et al., 2015; Kettering et al., 2013; Wang et al., 2006). There are several possible explanations for this result. One is that the basal fertilizer was mainly in the surface layer and the plastic film prevented any N leaching by rainfall (Wang et al., 2015; Zhang et al., 2012; Locascio et al., 1985; Ruidisch et al., 2013); secondly, the rate at which nitrate-N is transported with soil water to the soil surface layer will increase with a higher soil temperature under the plastic film mulching (Jiang et al., 2016); and lastly, that the plastic film mulching improved the soil water and temperature, increasing soil microbial activity and enhancing the soil mineralization rate (Wang et al., 2006).

Generally, two peaks of inorganic N in RM-R were observed in the 0–10 cm soil layer during the maize growing season. One occurred after fertilizer application, and the other during crop maturity and harvest stages. The high soil inorganic N during the later stage of the maize growing season, was probably explained by the low nutrient requirement for crop growth at that stage and by enhanced soil mineralization caused by the higher temperature (Zhang et al., 2012; Standford et al., 1973; Wang et al., 2006). There was a large decrease in inorganic N content in the 0–30 cm soil layer after June in RM-R, because of the high maize growth and uptake of soil N during this period.

There were also two peaks in inorganic N in the top soil layers (0–30 cm) for RM-F. The first was caused by fertilizer application, but the peaks in June were probably caused by the lateral movement of N from the ridge due to lateral water movement (however, in 2013, it might also have be caused by the application of the remaining 30% of the N fertilizer). A large decrease (from approximately 100 mg kg^−1^ to less than 20 mg kg^−1^) occurred in the inorganic N content of the top soil layers following several rainfall events in RM-F and peaks occured in the deeper soil layers (60–100 cm) after storms (e.g. following a rainfall event of 120.8 mm in 22 July, 2013), especially in the treatment with the higher N application rate. This is most likely because the plastic film intercepted rainfall, which then flowed from the ridges into the furrows, enhanced the leaching of inorganic N, especially nitrate, from the top to deeper soil layers (Wang et al., 2015). Kettering et al. (2013) used ^15^N to trace the fate of soil N and found that nitrate leaching in RM mainly occurred in the furrows, especially at high N application rates, consistent with the observations in our study. Thus, consideration of appropriate N application rate is needed in RM systems to avoid nitrate leaching from the furrows.

In F treatments, the inorganic N increased following fertilizer application, then decreased during maize growth for top soil layers (0–30 cm); however, an increase was observed during the later stage of the maize growing season in deep soil layers (60–100 cm) at the higher N application rate, due to N leaching during rainfall.

### 3.3 Inorganic nitrogen pool

**Fig. 8:**
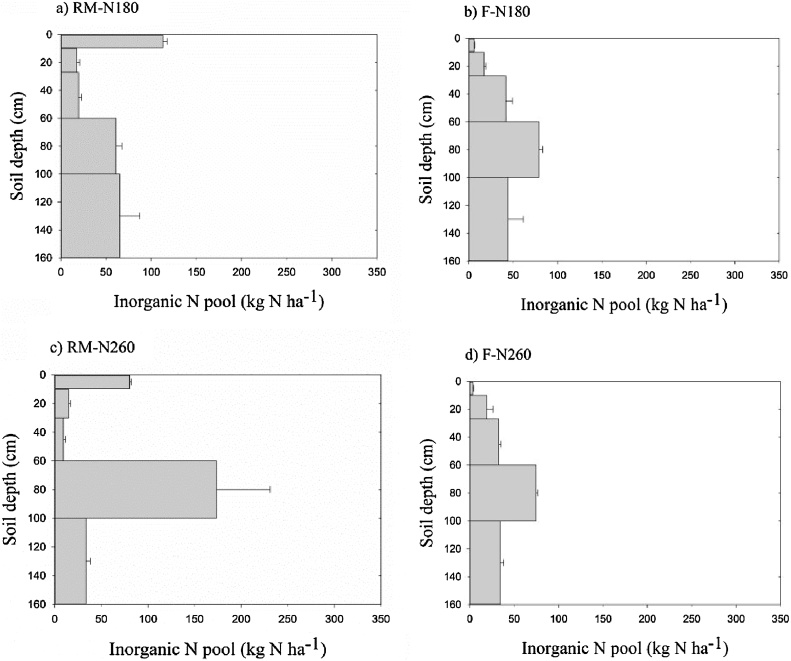
Inorganic N pools in a) RM-N180, b) F-N180, c) RM-N260, and d) F-N260 plots after one year of maize cultivation.

**Fig. 9:**
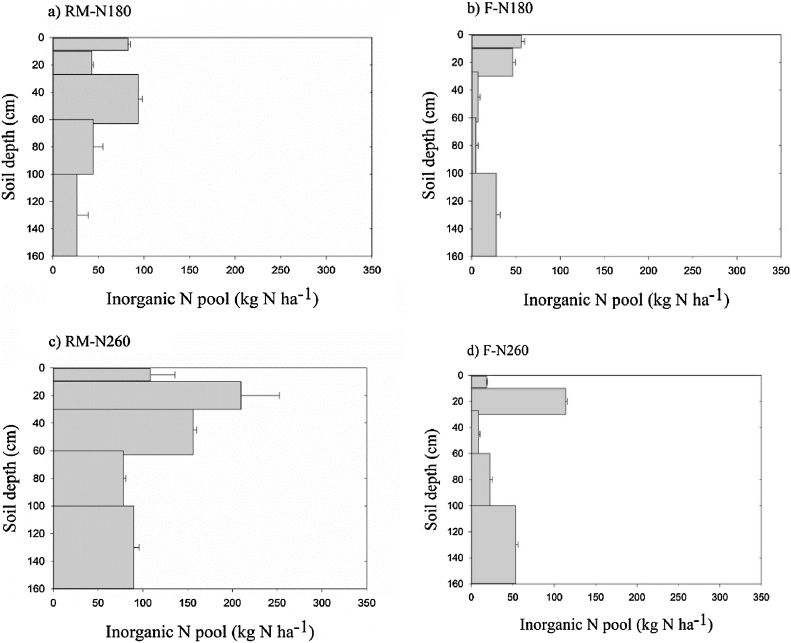
Inorganic N pools in a) RM-N180, b) F-N180, c) RM-N260, and d) F-N260 plots after two years of maize cultivation.

**Fig. 10:**
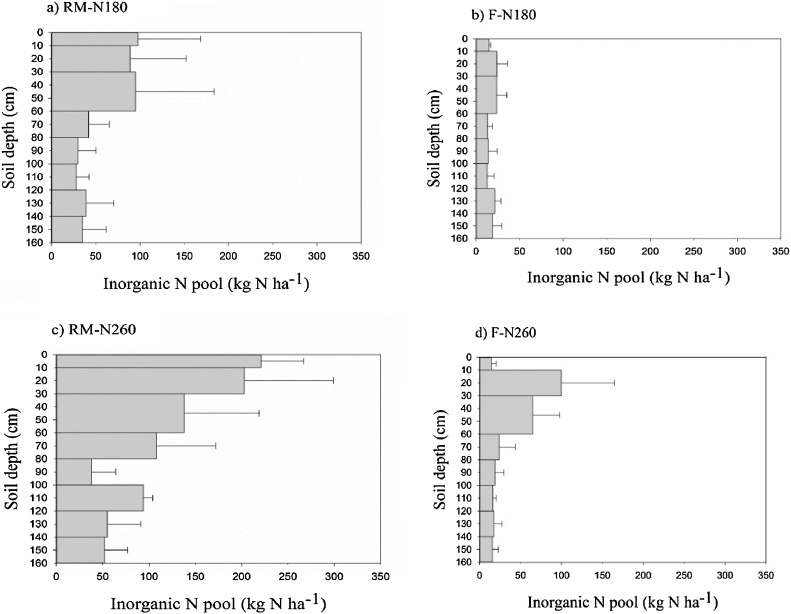
Inorganic N pools in a) RM-N180, b) F-N180, c) RM-N260, and d) F-N260 after three years of maize cultivation.

Inorganic N pools in the 0–10 cm and 60–100 cm soil layers were significantly higher than in other soil layers for RM after one year of maize cultivation. The two layers accounted for 26% and 41% of the total inorganic N pool (0–160 cm) for RM-N180 and RM-N260, respectively. About 40% of the total inorganic N was stored in the 60–100 cm soil layers in F treatments (Fig. 8). However, the large pool changed to the 0–60 cm soil layer in RM and to 0–20 cm and 100–160 cm soil layers in F after two and three years of maize cultivation (Fig. 9, Fig. 10). About 60% of the total inorganic N was present in the top soil layers (0–60 cm) after three years in RM treatments. The results indicate that soil inorganic N is likely to accumulate in top soil layers in RM but in deeper soil layers in F, implying that RM would reduce nitrate leaching during the maize growing season (Ruidisch et al., 2013; Zhang et al., 2012; Locascio et al., 1985). However, as we stated above, nitrate leaching from the furrows in RM at the high N application rate is of concern, which may cause nitrate accumulation in deep soil layers in Loess soil (Zhou et al., 2016). In addition, the higher nitrate-N accumulated in the top soils may lead to higher N_2_O emission in RM (He et al., 2018).

**Fig. 11:**
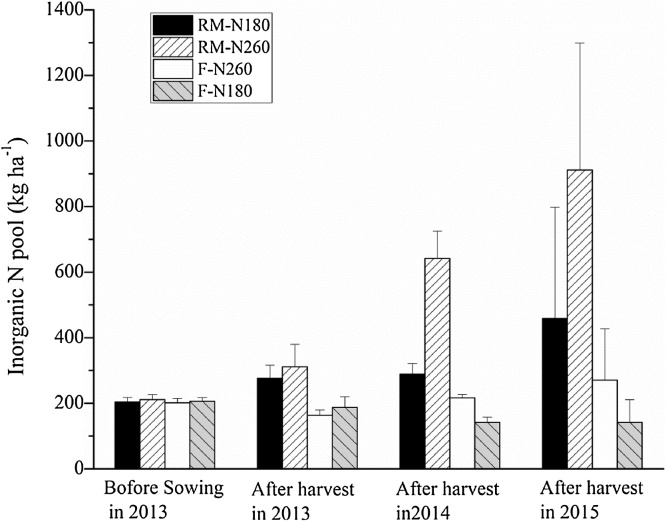
Inorganic N pools in the 0–160 cm soil layer before and after maize cultivation.

Inorganic soil N pools were initially small (200 kg N ha^−1^), but under RM increased significantly, by two and three times for RM-N180 and RM-N260, respectively, after three years of maize cultivation (Fig. 11). Thus, RM can enhance inorganic N accumulation in the soil profile, especially at high N application rates (Liu et al., 2014a; Kettering et al., 2013). The high inorganic N accumulation in our study was probably caused by higher soil mineralization in RM (Zhang et al., 2012; Hai et al., 2015). However, several studies have highlighted the many differences which may occur between the mulching system compared with the traditional system, such as soil temperature, water content, plant uptake, microbial activity, mineralization rate, and soil organic carbon content (Zhang et al., 2012; Liu et al., 2014a; Liu et al., 2014c; Jiang et al., 2016). These differences may interact to stimulate the release and accumulation of inorganic N in soil under RM; however, the mechanism is not clear so far. The total inorganic N pools in F were a slight decrease for F-N180 and a slight increase for F-N260 after the three years of maize cultivation. The soil water in F was not limiting in 2014 and 2015, which was good for plant growth and thus increased plant N uptake or caused N leaching and may have reduced the soil N accumulation.

The N application rate of 180 kg N ha^−1^ was as recommended for traditional maize cultivation in the study area, which was proven to be appropriate by our study, with a larger partial factor productivity (PFP) for RM-N180, ranging from 75% to 91%. The treatments at the higher N application rate of 260 kg N ha^−1^ had lower PFP values, even under RM. Across the three years, with the increase in the inorganic N pool, the PFP values in RM increased in 2014 but decreased in 2015. Cui et al. (2008) showed that residual soil nitrate in the top 100 cm of the soil profile should be maintained within the range of 87–180 kg N ha^−1^ for high yield maize production. This indicated that although maize grain yield has a positive relationship with N input, high N input or high residual soil N could reduce N use efficiency and limit the grain yield (Liu et al., 2017a, Liu et al., 2017b), probably also being related to soil organic carbon depletion. Except for in the F-N180 treatment, the ratio of inorganic N to TN increased sharply after three years of maize cultivation, with the largest increase occurring in the RM-N260 treatment, (1.7%–7.8%, Table 4). In the RM-N180 treatment, although inorganic N increased, the proportion as nitrate-N decreased after two and three years cultivation. These results suggest a higher nitrate leaching risk in RM-N260. However, although RM-N 180 resulted in higher PFP value and lower nitrate leaching risk, the high inorganic N accumulation in the soil profile after two/three years cultivation may lead to subsequent reductions in grain yield. It may also pose threats to the environment (increasing N leaching and greenhouse gas emissions) or cause the depletion of soil organic carbon (Liu et al., 2013), thus negatively affecting agriculture sustainability. Consequently, our results showed that N application rate of 260 kg N ha^−1^ was excessive in RM and the recommended N application rate of 180 kg N ha^−1^ for maize under traditional cultivation in this rain-fed area may not be suitable for RM. Liu et al. (2014b) found that an N application rate of 110 kg N ha^−1^ could sustain high grain yields in a fully mulched ridge and furrow system. Therefore, further studies to trace the fate of applied fertilizer N, investigate the soil mineralization and derive recommendations for N application rates under RM systems are warranted.

## 4 Conclusion

Implementing an RM mulched system compared with traditional (flat) cultivation significantly changed the soil water and inorganic N distribution over the 3-year study period. Compared with F, RM improved the soil water storage significantly during the pre-silking stage by decreasing the soil evaporation and increasing the plant transpiration, which improved the water use efficiency and contributed to enhanced grain yield. Higher SWS at sowing played an important role in maize production in rain-fed agriculture on the Loess Plateau, thus treatments such as plastic film mulching or straw residue left after harvest may be helpful in harvesting and storing rainfall in fallow season for subsequent use by the following crop. The soil inorganic N in RM accumulated in the top soil layer (0–10 cm) under the mulched ridge, probably because of reduced N leaching, increased soil mineralization, and lateral N movement from the furrow to the ridge. This was associated with a decrease in nitrate leaching to deeper soil layers. However, the nitrate leaching from the furrow was observed in the RM system at the higher N application rate. Inorganic N accumulation under the RM system increased by two to three times after three years of maize cultivation, which may be explained by enhanced soil mineralization. However, the mechanism for soil inorganic N accumulation under plastic film mulching systems is not clear, with potential interactions between a number of factors and soil processes, and further study is required. Overall, our result showed that RM-N180 could obtain high grain yield with low risk of nitrate leaching after one year of cultivation, but after three years of cultivation was associated with increased inorganic N accumulation in the soil profile. Thus, the recommended N application rate for traditional maize cultivation in this region may not be suitable under RM. Development of new recommendations for N fertilizer application rates under RM are needed to ensure the sustainability of rain-fed agriculture on the Chinese Loess Plateau.
